# Detection of *Ascaris lumbricoides* by Capsule Endoscopy

**DOI:** 10.4274/balkanmedj.galenos.2018.2018.1548

**Published:** 2019-02-28

**Authors:** Pavel P. Poliakov, Anar Y. Alimetov, Alexandr V. Onopriev, Andrey V. Avakimyan, Azamat Kh. Kade

**Affiliations:** 1 Department of Pathophysiology, Kuban State Medical University, Krasnodar, Russia; 2 Department of Postgraduate Surgery, Kuban State Medical University, Krasnodar, Russia; 3 Klinika-A OOO (Limited Liability Company), Krasnodar, Russia

A 47-year-old Caucasian woman complained of abdominal discomfort and general weakness. Her medical history indicated travels to ascariasis-endemic areas. Her hemoglobin level was 7.6 g/dL. Esophagogastroduodenoscopy revealed no evidence of active bleeding and colonoscopy revealed no pathology.

Capsule endoscopy revealed a live helminth in the terminal ileum, which was identified as *Ascaris lumbricoides* (Figure 1). Written informed consent was obtained from the patient. There were no adverse effects or complications of the procedure. Albendazole was prescribed to the patient.

*A. lumbricoides* is the most common soil-transmitted helminth, infecting more than 800 million patients globally (1). An increase in global migration and travel contributes to the prevalence of ascariasis, as well as other soil-transmitted helminth infections, in non-endemic regions. An absence of travel to endemic regions in an individual’s medical history does not exclude the possibility of ascariasis. This is because in addition to *A. lumbricoides*, humans can also be infected with *Ascaris suum*, which typically infects pigs (there are arguments in favor of these nematodes belonging to the same species) (1,2).

Three main pathogenic mechanisms underlie the numerous manifestations of ascariasis that resemble the symptoms of various diseases. Migration of larvae into the lungs damages the blood-air barrier (causing hemoptysis) and induces type I hypersensitivity reaction (eosinophilia and Löffler syndrome). Larvae mature into adult helminth in the intestine resulting in abdominal pain, damage to liver and pancreas, and malabsorption. Asymptomatic cases are also common (1,2,3,4).

Soil-transmitted helminth infections should be considered when conducting differential diagnosis in patients with concealed gastrointestinal bleeding and anemia that cannot be explained by other causes (2,3). In such cases, capsule endoscopy offers the ability to visualize the intestinal mucosa.

## Figures and Tables

**Figure 1:**
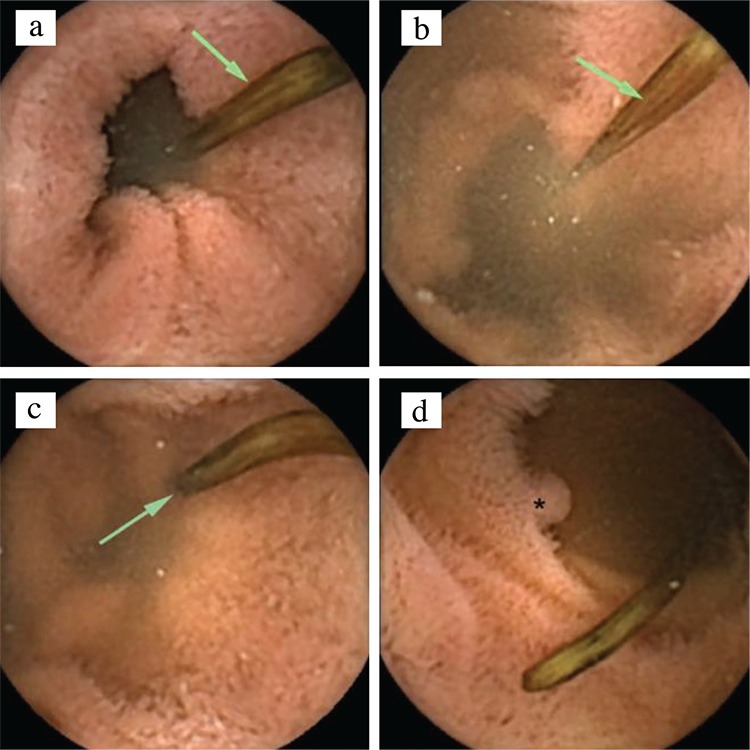
Live helminth (arrow) in bowel lumen (a-c). Pseudopolyp (asterisk) (d).
